# No prescription? No problem: drivers of non-prescribed sale of antibiotics among community drug retail outlets in low and middle income countries: a systematic review of qualitative studies

**DOI:** 10.1186/s12889-021-11163-3

**Published:** 2021-06-03

**Authors:** Sewunet Admasu Belachew, Lisa Hall, Daniel Asfaw Erku, Linda A. Selvey

**Affiliations:** 1 School of Public Health, The University of Queensland, 288 Herston Rd, Herston, Qld 4006 Australia; 2 School of Pharmacy, Faculty of Medicine and Health Sciences, University of Gondar, Gondar, Ethiopia; 3 Centre for Applied Health Economics, School of Medicine & Menzies Health Institute Queensland, Griffith University, Griffith, Queensland Australia

**Keywords:** Antibiotic dispensing, Driver, Factor, Low and middle income countries, Non-prescription, Pharmacy

## Abstract

**Background:**

Non-prescription dispensing of antibiotics, one of the main sources of antibiotic misuse or over use, is a global challenge with detrimental public health consequences including acceleration of the development of antimicrobial resistance, and is facilitated by various intrinsic and extrinsic drivers. The current review aimed to systematically summarise and synthesise the qualitative literature regarding drivers of non-prescribed sale of antibiotics among community drug retail outlets in low and middle income countries.

**Methods:**

Four electronic databases (PubMed, CINAHL, Scopus and Google Scholar) and reference lists of the relevant articles were searched. The Joanna Briggs Institute’s Critical Appraisal Checklist for qualitative studies was used to assess the quality of included studies. The enhancing transparency in reporting the synthesis of qualitative research statement was used to guide reporting of results. Data were coded using NVivo 12 software and analysed using both inductive and deductive thematic analysis.

**Results:**

A total of 23 articles underwent full text review and 12 of these met the inclusion criteria. Four main themes were identified in relation to facilitators of non-prescribed sale of antibiotics among community drug retail outlets: i) the business orientation of community drug retail outlets and tension between professionalism and commercialism; ii) customers’ demand pressure and expectation; iii); absence of or a lax enforcement of regulations; and iv) community drug retail outlet staff’s lack of knowledge and poor attitudes about antibiotics use and scope of practice regarding provision.

**Conclusions:**

This review identified several potentially amendable reasons in relation to over the counter dispensing of antibiotics. To contain the rise of antibiotic misuse or over use by targeting the primary drivers, this review suggests the need for strict law enforcement or enacting new strong regulation to control antibiotic dispensing, continuous and overarching refresher training for community drug retail outlet staff about antibiotic stewardship, and holding public awareness campaigns regarding rational antibiotic use.

**Supplementary Information:**

The online version contains supplementary material available at 10.1186/s12889-021-11163-3.

## Background

Antibiotics are among the most commonly prescribed medicines globally, and their consumption is increasing, in just 15 years (2000–2015) the increase was 65% (21.1 to 34.8 billion daily defined doses) [1, 2]. The surge in consumption was reported to be primarily linked with the increase in the use of antibiotics across low and middle income countries (LMICs) [1, 2]. In LMICs, the consumption increased by 114%, from 11.4 to 24.5 billion defined daily doses between 2000 to 2015 years [1]. According to World Bank income classification, LMICs include low-income economies with a Gross National Income (GNI) of $1035 or less, lower-middle income economies GNI per capita between $ 1036 and $ 4045, and upper-middle income economies with a GNI per capita between $4046 and $12,535 [3]. The consumption rate of antibiotics reported to continuously increase in LMICs [1, 4], partly driven by economic growth and prosperity [5]. However, the antibiotic provision and use in majority of these countries has often been irrational [6].

The relationship between irrational antibiotics use and development of antimicrobial resistance (AMR) is clear [7–9]. The consequences of AMR are multi-level, affecting the population’s health and the economy. It is estimated that by 2050, nearly 10 million deaths worldwide will be attributed to antimicrobial resistant infections and could cost the world up to USD 100 trillion if action is not taken [10]. Recently, in 2017, the World Bank report also noted that in the low and high AMR-impact scenario compared to no AMR effects, the world will lose 1.1 to 3.8% of its annual GDP by 2050, respectively [11]. It is reported that the highest toll of AMR [12, 13] and its worst impact occurs in the developing world [14], attributed to the relatively poor healthcare system coupled with the weak economy [4]. The causes for the development of AMR are complex and multifaceted, and the overuse/misuse are among the key contributors [15]. In this respect, over the counter (OTC) supply of antibiotics from retail outlets remains a major practice fuelling the increase of AMR in the world, even more so in the developing world [6, 16, 17]

In most LMICs, patients can easily obtain antibiotics without prescription from their families, relatives and/or nearby community drug retail outlets (CDROs) [18]. Among the different channels from which patients in LMICs can access antibiotics, CDROs have been identified to be a predominant source of antibiotics for the wider population [19–22]. The non-prescription sale of antibiotics is profoundly common practice in LMICs as demonstrated by the high prevalence of the practice identified in simulated clients and questionnaire based surveys [16, 17, 22–33]. This non-prescription sale of antibiotics is common, despite regulation in many LMICs making non-prescription supply of antibiotics illegal [6]. CDRO’s staff are the main actors facilitating the sale of antibiotics for different groups of customers whenever requested and often suggest or dispense antibiotics for visiting clients. In this respect, the motive for such mal-practice usually varies from place to place attributed to the difference in regulatory framework, economy and other socio-cultural factors across countries [17]. Because of this, a single factor claimed for a certain place could not be directly inferred for the other.

It is important to understand why CDRO staff dispense antibiotics to patients or clients without a valid prescription. Such evidence could help policy makers, law enforcers or other stakeholders, to target action and policy optimally. This could further assists efforts to design and propose an effective sustainable interventional strategy to transform CDROs in to lifetime antibiotic stewards. In this respect, evidence from qualitative studies is useful, as these studies are designed to identify specific determinants of antibiotic use, as they answer highly relevant question “why do pharmacy workers dispense antibiotics without prescriptions?”, and are preferred research approaches in developing concepts or theories for potential quantitative research, therefore, compiling qualitative evidence will be more beneficial in responding to the main research question.

The current review aims to synthesise and describe existing qualitative evidence about the reasons why antibiotics are sold as OTC drugs at CDROs in LMICs. To date, we have not found a review building on existing literature published in LMICs. One review was completed globally to identify the determinants of non-prescription antibiotic sale with the main focus on quantitative findings [34], however the study lacked specific recommendation for LMICs. The review was limited to a few qualitative studies from LMICs published till 2017. The current review focuses on qualitative studies since the methodologically robust approach to address the main research question is qualitative study, therefore, the information that will be generated through this review will be more valid as it has utilised studies that has been conducted using qualitative approach. In addition, the current review and the previous one [34] will complement each other in generating complete evidence around the topic as the previous one focuses more on quantitative studies. Therefore, with the current qualitative review, we hope to generate new and informative data about the subject in LMICs through an updated and more comprehensive synthesis of the available evidence.

## Methods

The current review was performed in accordance with the ‘enhancing transparency in reporting the synthesis of qualitative research’ (ENTREQ) guideline [35], and the study protocol was registered on PROSPERO (CRD42020203302).

### Data sources and search strategy

We adopted a broad search strategy to include all relevant qualitative studies. Using a previous study conducted by Belachew SA et al. as an input to set the data sources and search strategy [36], we did an electronic search of the following databases: PubMed, CINAHL, Scopus, and Google Scholar for qualitative studies that explored the reasons why antibiotics are sold without a valid prescription at CDROs. The key words used to retrieve the relevant articles were: (Driver* OR Reason* OR Factor* OR Determinant*) AND (“Dispense” OR sale* OR practice OR over the counter OR non-prescription OR “without prescription” OR “Self-prescribe” OR “self-treatment” OR “self-medication”) AND (“Community Pharmacy” OR “Drug store/shop” OR “private pharmacy” OR “Community Pharmacy professionals” OR “Druggist/Pharmacy technicians” OR “drug/medicine vendor/personnel. These were customised to each database. Searches were restricted to studies conducted in LMICs. The search included articles published in English from the inception of each database until the second week of May 2020.

Additional hand searches (references and citations of the included articles checked) were conducted to further trace eligible studies that were not retrieved in the databases search. Details on search terms and the number of records identified are provided in (additional file 1).

### Eligibility screening

The articles retrieved were then exported from Endnote X9 (EndNote X9 for Windows & Mac, released 28 April 2020) to COVIDENCE (Covidence systematic review software, Veritas Health Innovation, Melbourne, Australia) for screening and identification of articles to be included. All titles, abstracts and full texts were independently screened by two reviewers (SAB, DAE) to identify eligible studies, and discrepancies were addressed through mutual consensus among the two reviewers.

### Inclusion and exclusion criteria

Studies (including preprints, course completion papers/thesis) were included if they were, i) Qualitative studies that explored the reasons why the CDRO workers provide antibiotics similar to other OTC medicines without a prescription in CDROs, ii) Qualitative studies conducted among pharmacy or non-pharmacy professionals that reported the drivers for inappropriate antibiotics provision at CDROs, iii) Mixed methods studies which had a distinct section dedicated to qualitative data collection and analysis and reported data regarding the drivers for why antibiotics are being dispensed without prescription by CDRO workers. Studies were excluded if they were, i) studies that were published in languages other than English, ii) only abstracts with no full text available for retrieval, or were reviews, conference proceedings, letter to editor and meeting notes., iii) Studies not undertaken in LMICs.

### Quality assessment of the included studies

We used the Joanna Briggs Institute’s (JBI) Critical Appraisal Checklist for qualitative studies to assess the quality of included studies [37]. Two reviewers performed the quality assessment of the studies (SAB, DAE).

### Data extraction and analysis/synthesis

For all included full-text articles, one reviewer recorded the study characteristics on a customised spreadsheet and then this was cross-checked by the other reviewer. Adhering to the guidance from Thomas 2008 [38], quotes of study participants and main concepts summarising the relevant findings were extracted from the primary studies. During the search for the relevant information, the whole text included in the “results” section of the articles were assessed and coded. To assist the coding process, NVivo 12 version software (QSR International Pty Ltd. Australia 2020) was utilised. One reviewer coded the data (SAB) and this was reviewed by the second reviewer (DAE). Discrepancies were resolved through discussion.

We employed thematic synthesis as detailed by Thomas 2008 [38]. The synthesis followed three steps.

### Step one and two: line-by-line coding and identifying the descriptive themes

After a very careful read through of the included studies, key concepts were extracted and coding assigned to the data. For the coding, we used both deductive and inductive approaches. This allowed flexible data navigation to mark concepts that may not have been predefined. The concepts from the data were coded on line-by-line basis using NVivo software. After the completion of the coding, the researchers discussed on the upcoming findings and filtering of the coded data. Then, in the NVivo software, descriptive themes were developed by looking at similarities and differences between codes in the mother and child nodes.

### Step three: developing the analytical themes

In this final step of the synthesis, we organised/synthesised the descriptive themes/codes in to more abstract, overarching analytical themes to address review questions posed, and for better interpretation and discussion of the findings. The identified analytical themes fully comprised the descriptive ones. The implications we draw from this review were based on the analytical themes. In this study, the analytical themes are presented in the results section and also interpreted in the discussion.

## Results

### Description of studies

**Fig. 1:**
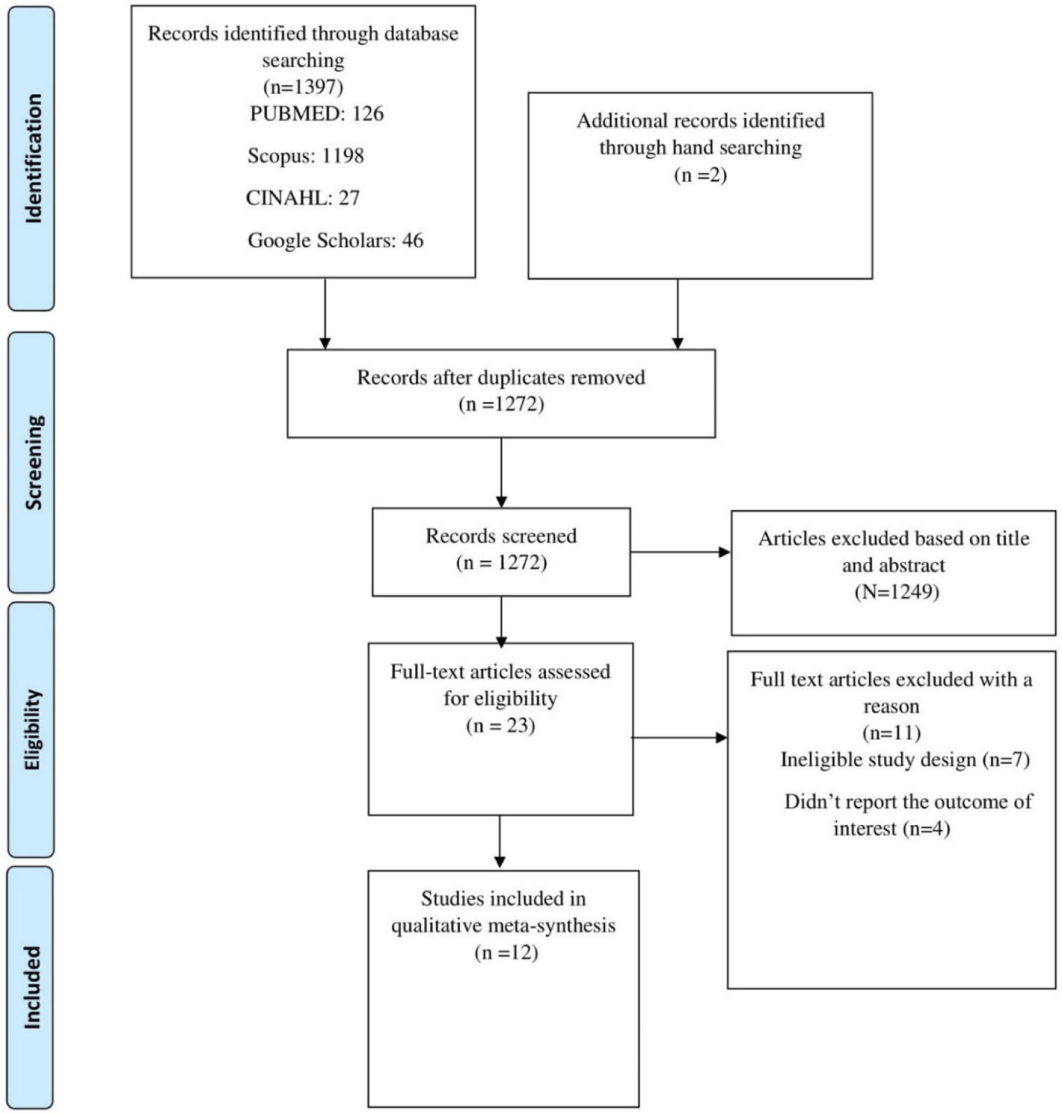
Flow chart showing selection of eligible studies for inclusion in the qualitative review

Four database searches and hand searching were completed in May 2020. After eliminating duplicates, 1272 studies with unique title and abstracts were identified. After initial screening based on title and abstract, 23 records retrieved were identified as relevant to undergo full text evaluation. Finally 12 studies conducted in nine LMICs were identified as eligible for inclusion in the final synthesis to generate evidence (Fig. 1).

All included studies were conducted in LMICs and all were qualitative studies. This review included 12 studies that took place in nine LMICs. More than half of the studies were conducted either in Sub-Saharan Africa (*n* = 4) [24, 39–41] or South Asia (*n* = 4) [42–45]. Most of the included studies instituted in depth or face to face interview (*n* = 10) [24, 38–42, 44–47] while the other two employed a focus group discussion (*n* = 1) [44] or a focus group discussion plus in depth interview (*n* = 1) [48] to explore the reasons for dispensing antibiotics without prescription or drivers for inappropriate dispensing of antibiotics. A total of 442 respondents participated in the included studies, ranging from five in Ethiopia [40] to 147 in Syria [46]. Across studies different interview guides were used (Table 1). However, all studies included questions that explored the drivers of inappropriate antibiotic provision that includes OTC sale of antibiotics. All studies clearly outlined their study aims and defined their data sources (participants and/or recruitment sites).

**Table 1:** Characteristics of the included studies

Citation	Country	Data collection methods	Sample size	Participants	Study purpose
Erku et al. (2018) [24]	Ethiopia	In depth-interview	13	Pharmacist/ Pharmacy assistant	To examine motivations behind dispensing antibiotics without prescription
Gebretekele et al. (2016) [40]	Ethiopia	In depth-interview	5	Pharmacist/ Pharmacy assistant/druggist	To explore reasons for OTC sales of antibiotics
Salim et al. (2017) [41]	Sudan	Face to face interview	30	Pharmacist	To explore reasons for non-prescription sales of antibiotics
Dillip et al. (2015) [39]	Tanzania	In depth-interview	84	Dispensers-owner and only dispenser	To explore the motivations for antibiotic dispensing
Bahnassi et al. (2015) [46]	Syria	Semi-structured face to face interview -	147	Pharmacist	To explore attitudes and practices in regard to antibiotic dispensing without prescription
Asghar et al. (2020) [42]	Pakistan	In depth interview	16	Pharmacy workers	To investigate the reasons that drive the inappropriate dispensing of antibiotics
Barker et al. (2017) [43]	India	In depth- Interview	20	Pharmacy workers	To explore factors that drive inappropriate antibiotic dispensing
Nga et al. (2014) [48]	Vietnam	Focus group discussion and in-depth interview	6	Pharmacy staff	To explore factors that impact inappropriate antibiotic dispensing
Kotwani et al. (2014) [44]	India	Focus group discussion	40	Pharmacist	To understand the antibiotics dispensing practices and behaviour
Kotb et al. (2018) [47]	Egypt	In depth-interview	25	Pharmacist	To explore the underlying causes of the sales of antibiotics without prescriptions
Saleem et al. (2019) [45]	Pakistan	In depth-interview	12	Pharmacist	To explore the determinants of AMR and the pattern of antimicrobial dispensing
Wang et al. (2020) [49]	China	In depth-interview	44	Key informants (pharmacists, FDA officials, residents)	To explore the determinants of non-prescription dispensing of antibiotics

### Quality assessment of the included studies

Quality and risk of bias assessment for all of the included studies were conducted using the 10 criteria of JBI Critical Appraisal Checklist for qualitative studies. Nearly all (*n* = 10) [24, 39–43, 45, 46, 48, 49] met seven or more of the 10 JBI appraisal checklist criteria for qualitative studies. However, all of the included studies lacked clarity in terms of researcher reflexivity or details of interview questions. One of the included studies was conducted in Syria involving 147 participants, the study employed a semi-structured interview which consisted of both open and closed ended interview questions, [46],however the number of interviewee were high in number and the time spent for each interview was relatively short and this might have potentially affected the depth of the interview. Details of the quality assessment for the included studies provided as an additional file 2.

Building on the existing literature, we identified four recurring analytical themes: 1) the business orientation of CDROs and tension between professionalism and commercialism; 2); customers’ demand pressure and expectation; 3) absence of or a lax enforcement of regulations; and 4) CDRO staff’s lack of knowledge and poor attitudes about antibiotics use and scope of practice regarding provision. The themes identified, along with representative quotes are summarised below.

### Theme 1: the business orientation of CDROs and tension between professionalism and commercialism

The tension between the altruistic, service-oriented nature of health professionals (such as pharmacists) and the business-oriented, profit-driven environment of CDROs puts pressure on CDRO staff. The health professional–retailer nexus is particularly more pronounced among CDROs in LMICs where CDROs are often confined to the traditional roles of selling medicines and their involvement in service delivery other than dispensing (e.g. patient education and counselling, early detection and referral of patients with chronic illness, etc.) is insignificant. Findings from this review suggest that CDRO  staff often prioritise commercial rather than professional aspects of their profession by dispensing antibiotics without a prescription.

CDRO staff are often caught in between dichotomy of roles including both retailing and healthcare provision. Working in a CDRO retail environment, such ethical quandaries are common and in circumstances where either one of these roles are to be prioritised, the choice comes down to, or is at least influenced by, owner’s and/or manager’s discretion on what to do. This notion of being influenced and pressured by CDRO owners to sell antibiotics if requested, even if it is without a prescription, was reiterated across studies [24, 38–41, 46]. A study also mentioned that the CDRO owners give incentives or commissions to the staff based on the number of sales made while on duty [45].

*“Sometimes people start forcing. We are also pressurized by the owner of a pharmacy. If we refuse, customers go to another pharmacy and get medicine from there. It causes us business loss” (pharmacy worker)* [42]*.*

*“Reasons from my point of view are mainly financial; the owners of pharmacies want to increase their revenues” (pharmacist)* [41]*.*

“*… … ..I used to dispense medicine including antibiotics for common disease conditions to patients. At the end of the month, I get a good incentive based on the sale” (pharmacist)* [45]*.*

Since CDROs are run on a profit basis and are not subsidised by governments, strategies and activities that are in place are built on raising sales and increasing profit margins, and this includes continuous provision of antibiotics, which are the best-selling group of drugs in these countries, with or without a prescription [39, 40]. This leaves CDRO staff little time and incentive to engage in providing essential primary healthcare service to the public. It also makes it difficult to uphold the moral and ethical principles under which their profession is based [39, 40].

*“Every professional knows non-prescription sale of antibiotics is illegal and unethical practice. But the practice continues as professionals’ main interest is to maximise their profit” (participant/CMP002/)* [39]*.*

*“They want to make money, raise income … .that is the reason referrals are not provided” (dispenser-not owner)* [39]*.*

A fierce competition among rival CDROs to retain as many customers as possible was also cited as a reason for non-prescribed sale of antibiotics. It was noted that if CDRO staff in one CDRO refrain from dispensing antibiotics without a prescription, customers will go on to the next CDRO and get it easily, without a valid prescription. Such inconsistency in dispensing practice will, in the long run, cost those CDROs that are adhering to the ethical and regulatory standards by losing trust of customers and eventually hurting their business [24, 40, 42, 46–49]. In such environments where customer satisfaction or need is of a central value, most CDRO  staff opt to respond to customer’s request and need without properly scrutinising the consequences or repercussion of their actions [24, 39, 40, 42–44, 46–48].

*“It is very simple. If I am not happy to sell them (customers) these products, they will go to the neighbouring pharmacy and easily get them easily without a question. So it becomes a matter of competition and retaining customers”” (participant /p11/)* [24]*.*

*“My customers can get antibiotics anywhere, I cannot stop them, otherwise I will lose my customers” (pharmacist)* [46]*.*

*“I need to satisfy clients’ demand. That’s in the interest of our business!” (respondent)* [48]*.*

A study also mentioned that pharmaceutical representatives visit CDROs to advertise their products including antibiotics, and lobby staff to dispense whenever demanded through convincing them that they can make profit from selling antibiotics [45].

*“I think they have a negative effect. They usually push us to sell their product. I think that is not very good practice” (pharmacists)* [45]*.*

In one study, CDRO staff mentioned that they provided antibiotics with other products like Traditional Chinese Medicine with a primary intention of promoting and selling the Traditional Chinese Medicine products that are believed to earn more profit than antibiotics [49].

*“We do not make many profits from selling antibiotics. The price of a box of ciprofloxacin hydrochloride is only 10 RMB ($1.50). However, we use antibiotics mainly to promote the selling of Traditional Chinese Medicine, which is more expensive and brings more profit.” (pharmacist and owner)* [49]*.*

### Theme 2: customers’ demand pressure and expectation

This commercial-oriented practice and the notion of customer satisfaction is two-fold, and the relationship is one that is reciprocal and based on a win-win strategy: the CDRO owner wants to increase profit margins, and customers need access to antibiotics to treat their minor conditions although their conditions do not necessarily warrant an antibiotics. Some customers prefer and often opt to directly purchase antibiotics from CDROs as they strongly rely on their previous experience with the disease and treatment [39–41, 45–47, 49], that it would give them a quick relief. Moreover, customers do so for a number of other reasons including if they feel like their condition is not severe enough to visit a doctor [48], if they cannot afford to pay for consultation fee for physicians [39–42, 44, 45, 47, 49], or if they experience poor satisfaction with the services of health facilities in nearby areas (e.g. prolonged waiting time due to the overcrowded services, doctors unable/unwilling to have enough consultation time with patients and less quality service) [40–42, 47–49] or if there are no health facilities in nearby areas at all [41–43].

*“… .most of the customers questioned the necessity of going to clinic as they expect same antibiotic will be prescribed again and hence they opted to buy the same antibiotic directly from the pharmacy” (participant/CMP004/)* [39]*.*

*“I think many patients cannot afford consultation fees. We tried to refer them to clinics. However, they used to explain that they cannot afford consultation fees” (pharmacist)* [41]*.*

*“It’s very annoying and time-consuming to be examined in a hospital. And private clinic are very costly, as they do many kinds of test. Our customers only go to see doctors in case of severe disease” (both urban and rural respondents)* [48]*.*

*“Inaccessible health facilities are among the reasons that discourage patients from going to hospitals. We know that patients perceive us as a more convenient option” (pharmacist)* [41]*.*

In one study, CDRO staff mentioned that customers trust them as professionals and that they expect complete healthcare provision from CDRO staff [39].

*“Peoples trust us a professional, so when you give referral, they question your ability and you status might go down … besides we have all medicines to treat severe pneumonia” (owner and dispenser)* [39]*.*

### Theme 3: absence of or a lax enforcement of regulations

Absence of regulations or a lax enforcement of existing regulations regarding non-prescription sale of antibiotics further reinforced the practice of supplying antibiotics without a valid prescription. CDROs reported bypassing the law, if there is one, without being fined, and they do so with the purpose of increasing profit margins and retaining customers who are unable or unwilling to visit health facilitates to get examined and get a prescription [39, 40, 46, 49]. In addition, there is insufficient capacity to enforce existing legislation [49].

*“There is an absence of implemented policies that regulate the dispensing of antibiotics at community pharmacies” (pharmacist)* [41]*.*

It is also reported that the enforcement of the regulatory systems are not rigorous enough to identify CDROs that employ different mechanisms to get around regulatory checks. This can include presenting inappropriately acquired prescriptions from the private clinics or using fake prescriptions such as blank prescription sheets in order to justify the already issued antibiotics and sales records. Some also refuse to provide receipts to the clients [49].

### Theme 4: CDRO staff’s lack of knowledge and poor attitudes about antibiotic use and scope of practice regrading provision

CDRO staffs’ poor knowledge about the consequences of antibiotics misuse and/or the importance of rational antibiotic use were among the stated reasons for non-prescription antibiotic provisions. Some CDRO staff lacked knowledge and awareness on the impact that imprudent use of antibiotics has on the development of AMR [40, 44, 46], and also reported that CDRO staff are not well aware of the burden associated with the non-prescription antibiotic dispensing [40]. Some staff also think that dispensing antibiotics without prescription has become a common practice and they do not consider it as harmful or unlawful practice [38]. In addition, CDRO staff reported lack of knowledge regarding the regulatory frameworks governing antibiotic dispensing and/or being misinformed about these policies (e.g. assuming some types of antibiotics are permitted to be sold as OTC medicines) [48].

*“Some kind of weak antibiotics such as amoxicillin or ampicillin can be sold without prescription” (rural seller)* [48]*.*

Professional incompetence or poor knowledge as a result of lack of compulsory professional training especially among those who are not registered pharmacists (such as pharmacy assistants) and absence of continuous professional development materials or trainings contributed to the malpractices [42]. In addition, CDRO staff also lacked the necessary knowledge or competency for pharmacy practice as there are several untrained employees or non-pharmacy professionals working part-time at the CDROs [43].

*“No training is required for the pharmacy worker job. On job we learn how to read prescriptions and about medicines from our seniors. As seniors are also not professionals so lack of professionalism results in inappropriate dispensing of medicines” (pharmacy worker)* [42]*.*

The attitude or belief of CDRO staff towards antibiotic use or provision affected the pharmacy practice in CDROs*.* In one study, CDRO staff indicated that they feel more ease to provide antibiotics without prescription for a family member, thinking that they can easily follow up and trace them when (if) necessary [46]. CDRO staff also believed that refilling antibiotics, even if it is without a prescription, will not create a problem as patients have already been using it [46].

*“When a family member asks for an antibiotic, I do have adequate experience to dispense the one they need” (pharmacist)* [46]*.*

*“Sure, if they used it before that means they can use it again with no problem” (pharmacist)* [46]*.*

Across many studies, it was stated that some CDRO staff did not clearly distinguish their scope of practice or role, perceive as they have expanded role that encompasses diagnosing and treating patients [38, 40–45]. In addition, the CDRO staff assume that they have already acquired the necessary knowledge for assessing the patient and providing treatment/antibiotics [42, 46].

*“It is a part of our role as pharmacists to diagnose minor infections and to dispense antibiotics accordingly” (pharmacist)* [41]

## Discussion

Antibiotics are classified in many developing countries as prescription only medications and should only be handed over to a client up on presentation of a valid prescription. Despite this, CDROs across countries are still selling antibiotics without prescription. In this review, we systematically organised and synthesised published evidence on the reasons that leads CDROs to sell antibiotics without a valid prescription in LMICs. The contribution of each of these factors/reasons towards non-prescribed sale of antibiotics varies considerably from country to country owing to difference in underlying contexts.

CDROs in countries that have weak health systems and underdeveloped mechanisms for routine monitoring of medicines often take this as an opportunity to profit from the pharmaceutical transaction potentially compromising the quality of pharmaceutical care. The high business interest of CDROs often motivate CDRO owners to open CDROs so as to primarily serve their commercial interests [50]. The onerous and expensive nature of setting up a CDRO in low income setting drives CDROs to be business centred facilities with intention to compensate expenses and associated loans. In this regard, small-scale business loans with very low interest rates offered to pharmacists to facilitate setting up pharmacies were found to improve pharmacy practice [50]. Furthermore, medications are among highly valued goods, therefore, failure to regulate drug pricing could result in many healthcare professionals prioritising profit as there is no control over the drug selling price. Ethiopia, for instance, lacks a strong pharmaceutical pricing policy that enables monitoring of drug prices [51] which saw the opening of many CDROs. To mitigate this, prohibiting the offer of sales commission to CDRO workers have been suggested as one strategy in addition to the legislative measures [51]. Although understanding the nature and drivers of such pharmaceutical corruption and professional malpractice is beyond the scope of this study, this is undoubtedly one of the manifestations of the broader structural problems of healthcare systems in these countries.

For clients with low economic status who cannot afford to pay for physician consultations and associated diagnostic investigations, direct purchase of antibiotics from CDROs is a cheaper option as there is no consultation fees and are often open to negotiate drug options based on the customers’ financial capacity [6, 17]. Moreover, unavailability and/or inaccessibility of healthcare facilities in nearby places also propels patients to look for antibiotics directly from CDROs. Although improvements in access to health care have been reported in LMICs, significant portion of their community have limited access [52]. For instance, access to healthcare facilities remains a big challenge in Ethiopia [53] with more than half of the population in rural portion of Ethiopia lives more than 10 km from the nearest health facility, often with no access to public transportation facilities [54]. In addition, clients’ previous experience of recovering from an infection when taking certain antibiotics tend to boost their confidence to ask similar medication in other times. Yet, the disease may not be similar, or the previous therapy may not be appropriate, and similar symptoms could be caused by different illnesses. For instance, while a cough may be caused by a cold or allergies, it could also be related to a more serious problem like emphysema or congestive heart failure [55].

CDROs’ antibiotic dispensing practices were also reported to be partly influenced by pharmaceutical companies or product promoters. Promotions from companies with potential financial conflict of interest may convey biased information emphasising the benefits of the drugs being promoted and down playing the harms the drug causes if used inappropriately [56]. For instance, a study conducted in India by Thawani et al. revealed that local pharmaceutical companies and multinational subsidiaries were inappropriately using the standing of the WHO to promote their products in an effort to enhance the drug acceptability, sale and reputation [57]. Absence of or a weak implementation of prescription only antibiotic dispensing policy is partly attributed to the lack of expert personnel who can execute the legislation. In China, for instance, increases in Food and Drug Administration scope of practice [58, 59] contributed to the shortage of experts and increased workload of Food and Drug Administration officials, which in turn compromised the enforcement of policies regulating antibiotics supply. For similar reason, in Ethiopia, many CDROs have been also taking advantage of the regulatory gaps and lack of legal repercussion to receive medicines from illegal market across borders [60]. Moreover, for mutual financial gain, it has been reported that CDRO staff and prescribers work together to circumvent regulatory supervision via, for instance, providing blank fake prescriptions having a name of a prescriber and stamp to keep it as a record to later justify their dispensing during inspection [49].

The drivers of non-prescription antibiotics sale identified in this review are diverse (i.e. at the level of customers, owners, sellers and regulation), and thus demands a multi-level, long-term and targeted strategies to address such malpractice. As our review highlights, there is a need for a stringent law enforcement or enacting a very strong regulation to control the irresponsible provision of antibiotics in CDROs, plus implementing a strict regulatory system could be useful to overcome more than just one driver. A number of studies conducted in Zimbabwe, Chile, Colombia, Brazil, Mexico and Korea in addition to a study completed in Saudi Arabia found strict enforcement of existing laws to be effective in containing the non-prescribed antibiotics provision [61–65]. In an effort to improve the implementation of law enforcement, we believe, much more emphasis needs to be given to rural places compared to urban areas, this is because the inspection of pharmacy practice is relatively absent or less regular in rural places as the regulatory bodies are usually located in urban places, and access to healthcare facilities is relatively scarce in rural places so that the public demand for medications/treatments from CDROs would be comparably high. In resource limited settings, although enforcement of law that restrict the provision of antibiotics without prescription is mandatory to contain the inappropriate use of antibiotics, it has to be considered that the restriction might compromise access to antibiotics/treatment in these places where the healthcare facility is believed to be limited especially in rural areas. Therefore, to reduce the side effect of restriction/law enforcement on access in such settings, it would be imperative that the nations need to strongly work on expanding healthcare facilities all over the country along with patient education about rational antibiotic use so that the community in the remote or rural areas will have access to proper healthcare with affordable price.

Facilitating access to healthcare in rural and resource limited areas and reducing barriers to attend (e.g. transport, bureaucracy etc.) have a direct implication in reducing inappropriate use of antibiotics (including sourcing antibiotics from CDROs without a prescription). One strategy to realise this is by achieving universal health coverage (UHC), thereby ensuring that all community members have access to the most accessible, quality and affordable (minimising the out-of-pocket expenditure) healthcare service for the public. The UHC movement would be a good strategy to reduce population high demand of antibiotics directly from the CDROs escaping expert consultations and diagnostic evaluations [66]. However, moving forward to UHC is not easy for a nation particularly for LMICs, it demands strengthening the health system in the country and also requires a strong financing structure that could potentially demand pooling funds from insurances such as social or community based health insurances to support UHC as evidenced by a review conducted in Africa and Asia [67]. Otherwise, given that access to the health care facilities and physicians are scarce in many resource limited settings, providing extensive training to CDRO staff about antibiotic stewardship and management of minor ailments would ensure access to and prudent use of antibiotics as the CDRO staff could be capacitated to treat certain infections based on the countries treatment guidelines at least in rural or remote places. Hence, policies in such settings need to give much emphasis at promoting judicious use of antibiotics than restricting antibiotics as the infectious disease burden has been known to be high and fatal.

If pharmacists trained properly regarding prudent antibiotic use, they can be part of the solution to overcome the global challenge of antibiotic resistance (ABR) and emphasised that training can enhance CDROstaffs’ active involvement in antibiotic stewardship practices [68]. In relation to improving antimicrobial stewardship practices, data regarding antimicrobial utilisation and antimicrobial resistance is critical as it provides benchmarks and identify locations for targeted interventions, in this regards, a study revealed the importance of incorporating technology enhancements, smartphone applications and social media platforms to maximise the antimicrobial stewardship practices as it has been partly implicated to facilitate antimicrobial utilisation and antimicrobial resistance data reports [69]. One important commentary also suggested introducing IT antibiotic surveillance systems in the supply chain and monitoring pharmacy practice using mobile technologies as a strategy to reduce the non-prescription sale of antibiotics. However, associated costs and implementation challenges would be the greatest concern especially in resource limited settings. Indeed, the current review noted that pharmacy staff knowledge to antibiotics use or dispensing has been variable, in addition, other study conducted in Albania also witnessed variable knowledge of community pharmacy staff towards antibiotics, with merely 13% declaring antibiotics as infective against viruses [70]. This tells that CDRO staff should be equipped with the necessary knowledge regarding the detrimental consequences of non-prescription supply of antibiotics and the terrifying surge of antibiotic resistant infections following the injudicious antibiotic provision. In addition, enhancing the presence of licensed pharmacists on duty and promoting chain pharmacies could be important as it is implicated to be associated with less non-prescription sale of antibiotics and quality practice [71].

Likewise, educating the community/patients regarding rational use of antibiotics through public campaigns could assist or would be one important complement to other strategies to reduce an intense demand of antibiotics for self-medication, and may improve the community awareness about rational antibiotics use in general. For example, a pre-and post-intervention study in Egypt revealed that antibiotic use awareness campaign significantly improved the caregivers or patients’ knowledge and attitude towards antibiotics use; after the educational intervention, the caregivers/adults were less likely to put pressure on doctors or pharmacists to prescribe antibiotics [72]. Evidence form developed nations, for instance, Europe also showed that public antibiotic use awareness campaigns resulted a fall in public antibiotic use of 6.5–28.3% [73]. In general, multi-sectoral and concerted approach is needed to promote judicious use of antibiotic which may include enforcement of laws prohibiting the non-prescribed supply of antibiotics, CDRO staff training, public education, and also development of strong pharmacy practice surveillance system [16, 71]. In addition, an article assessing the impact of law enforcement in reducing non-prescribed supply of antibiotics concluded that comprehensive multifaceted interventions would be the most likely effective approaches in addressing over the counter provision of antibiotics [74].

Our review also highlighted some areas in the current literature that warrant further research. Nearly all of the studies were conducted among CDRO staff working in the urban or administrative towns where the CDRO staff’s awareness and the authorities’ service inspection is relatively good. This tells us that drivers may not be similar between urban and rural CDROs [48, 75]. Therefore, the current findings could not represent the case in rural town as the practice highly varies between these two different places. Given the reported reasons associated with the practice potentially differ from place to place in a country, the drivers for the non-prescription supply of antibiotics in non-urban CDROs need to be investigated in future research.

### Strengths and limitations

To the best of our knowledge, this is the first review thematically synthesising qualitative evidence regarding the reasons why CDRO staff dispense antibiotics without a valid prescription in CDROs of LMICs. We have employed extensive search strategies not to miss articles. The review used thematic analysis which is a preferred approach to synthesise qualitative evidence. Despite the strengths, the review has limitations. The review may miss articles if not indexed in the included databases or published in languages other than English. Furthermore, the lack of published studies from many LMICs was another limitation of the review.

## Conclusion

This review explores the reasons why CDRO staff sell antibiotics without a valid prescription in LMICs. A number of potentially amendable reasons were identified in relation to OTC antibiotic dispensing. To avert the rise of AMR, it would be worthwhile targeting the associated drivers for the non-prescribed sell of antibiotics. In this context, this review highlights the need for strict law enforcement or enacting new strong regulation to control antibiotic dispensing, continuous and overarching refresher training for CDRO staff about professional roles in regards to antibiotic stewardship, and holding public awareness campaigns regarding rational antibiotic use. Furthermore, CDRO staff are well positioned to be antibiotic stewards to avert the rise of AMR given their location and easy accessibility, but their potential is largely untapped. In this respect, we hope the current review provides an insight to explore the opportunities and challenges to the implementation of antibiotic stewardship in CDRO setting.

## Supplementary Information

**Additional file 1:.** Search terms and strategy

**Additional file 2:.** Quality assessment of the included studies

## Data Availability

All data generated or analysed during this study are included in this published article.

## Abbreviations

AMR: Antimicrobial Resistance
CDROs: Community Drug Retail Outlets
ENTREQ: Enhancing transparency in reporting the synthesis of qualitative research guideline
GNI: Gross National Income
JBI: Joanna Briggs Institute
LMICs: Low and Middle Income Countries
OTC: Over the Counter
USD: United States Dollar
UHC: Universal Health Coverage
