# Supplementary material for: No prescription? No problem: drivers of non-prescribed sale of antibiotics among community drug retail outlets in low and middle income countries: a systematic review of qualitative studies
Source: BMC Public Health. 2021 Jun 3;21:1056. doi: 10.1186/s12889-021-11163-3 (PMC8173982; doi:10.1186/s12889-021-11163-3)
Supplement: Supplementary file 1 — Additional file 1:. Search terms and strategy [file 12889_2021_11163_MOESM1_ESM.docx]

**No prescription? No problem: Drivers of non-prescribed sale of antibiotics among community drug retail outlets in low and middle income countries: a systematic review of qualitative studies.**

Sewunet Admasu Belachew^1, 2*^, Lisa Hall^1^, Daniel Asfaw Erku^3^**,** Linda A Selvey^1^

^1^School of Public Health, The University of Queensland, 288 Herston Rd, Herston, Qld 4006, Australia

^2^School of Pharmacy, Faculty of Medicine and Health Sciences, University of Gondar, Ethiopia

^3^Centre for Applied Health Economics, School of Medicine & Menzies Health Institute Queensland, Griffith University, Queensland, Australia.

***Corresponding author**

Sewunet Admasu Belachew

School of Public Health, The University of Queensland, 288 Herston Rd, Herston, Qld 4006, Australia

Email: [s.admasubelachew@uq.edu.au](mailto:s.admasubelachew@uq.edu.au)

**Authors’ email address**

Linda A Selvey: [l.selvey@uq.edu.au](mailto:l.selvey@uq.edu.au)

Lisa Hall: [l.hall3@uq.edu.au](mailto:l.hall3@uq.edu.au)

Daniel Asfaw Erku: [d.erku@griffith.edu.au](mailto:d.erku@griffith.edu.au%20)

**Search terms and strategies**

**Date of search:** from inception to second week of May, 2020

**PubMed (126)**

| Search ((((((((((((((Reason*) OR Factor*) OR Motive*) OR Driver* OR determinant*)) AND ((((((agents, antibacterial[MeSH Terms]) OR antibacteria*) OR "antibiotic agent* " ) OR agents, antimicrobial[MeSH Terms]) OR antimicrobia*)))) AND (((((((sale[MeSH Terms]) OR Misuse* OR Overuse* OR Inappropriate OR Irrational OR non-judicious OR Self-medication* OR self-prescri* OR practice* OR Self-treatment* OR management* OR over the counter drugs[MeSH Terms]) OR dispens*) OR "without prescription" OR provision* OR Non-prescrib*) OR supply*))))) AND ((((((((community pharmacy[MeSH Terms]) OR Drug-store*) OR Drug-vendor* OR Drug-shop*) OR Retail-Pharmac*) OR Community-Pharmacist*) OR Druggist*) OR Pharmacy-technician* OR Pharmacy-personnel* OR Pharmacy-assistant* OR “community pharmacy service*”))))))) AND ((“Central Africa” OR Cameroon OR “Central African Republic” OR Chad OR Congo OR “Democratic Republic of the Congo” OR “Equatorial Guinea” OR Gabon OR “Sao Tome and Principe” OR “Eastern Africa” OR Burundi OR Djibouti OR Eritrea OR Ethiopia OR Kenya OR Rwanda OR Somalia OR “South Sudan” OR Sudan OR Tanzania OR Uganda OR “Southern Africa” OR Angola OR Botswana OR Eswatini OR Lesotho OR Malawi OR Mozambique OR Namibia OR South Africa OR Zambia OR Zimbabwe OR Africa, Western OR Benin OR “Burkina Faso” OR “Cabo Verde” OR “Cote d'Ivoire” OR Gambia OR Ghana OR Guinea OR Guinea-Bissau OR Liberia OR Mali OR Mauritania OR Niger OR Nigeria OR Senegal OR “Sierra Leone” OR Togo OR Afghanistan OR Bangladesh OR Bhutan OR Bolivia OR Cambodia OR Comoros OR Egypt OR “El Salvador” OR Haiti OR Honduras OR India OR Indonesia OR Kiribati OR “North Korea” OR “Kyrgyz Republic” OR Laos OR China OR Madagascar OR Micronesia OR Moldova OR Mongolia OR Morocco OR Myanmar OR Nepal OR Nicaragua OR Pakistan OR Philippines OR “Solomon Islands” OR Swaziland OR “Syrian Arab Republic” OR Tajikistan OR Tunisia OR Ukraine OR Uzbekistan OR Vanuatu OR Vietnam OR Palestine OR Yemen OR Libya OR Algeria OR Armenia OR Azerbaijan OR Belarus OR Belize OR “Bosnia and Herzegovina” OR Brazil OR Colombia OR “Costa Rica” OR Cuba OR Dominica OR “Dominican Republic” OR Ecuador OR Fiji OR Georgia OR Grenada OR Guatemala OR Guyana OR Honduras OR Iran OR Iraq OR Jamaica OR Jordan OR Kiribati OR Kosovo OR “Lao People's Democratic Republic” OR Lebanon OR Macedonia OR Malaysia OR Maldives OR “Marshall Islands” OR Mexico OR Paraguay OR Peru OR “Russian Federation” OR Samoa OR Serbia OR “Sri Lanka” OR “Saint Lucia” OR “Saint Vincent and the Grenadines” OR Suriname OR Thailand OR Timor-Leste OR Turkey OR Tonga OR Turkmenistan OR Tuvalu OR Vanuatu OR Venezuela OR “West Bank and Gaza” OR “Federated States of Micronesia” OR Moldova OR Mongolia OR Montenegro OR Myanmar OR Nauru OR Nicaragua)) Sort by: Best Match |
| --- |

**Scopus (1198)**

| ( ALL ( reason*  OR  factor*  OR  motive*  OR  driver*  OR  determinant* )  AND  ALL ( "antibacterial agent*"  OR  antibacteria*  OR  "antibiotic agent* "  OR  antibiotic  OR  "antimicrobial agent"  OR  antimicrobia* )  AND  ALL ( sale*  OR  misuse*  OR  overuse*  OR  inappropriate  OR  irrational  OR  non-judicious  OR  "Self medication*"  OR  "self prescri*"  OR  practice*  OR  "Self treatment*"  OR  management*  OR  "over the counter*"  OR  dispens*  OR  "without prescription*"  OR  provision* )  OR  ALL ( "non prescri" )  AND  ALL ( "community pharmac*"  OR  "Drug store*"  OR  "Drug vendor*"  OR  "Drug shop*"  OR  "Retail Pharmac*"  OR  "Community Pharmacist*"  OR  druggist*  OR  "Pharmacy technician*"  OR  "Pharmacy personnel*"  OR  "Pharmacy assistant*"  OR  "community pharmacy service*" )  AND  ALL ( "Central Africa"  OR  cameroon  OR  "Central African Republic"  OR  chad  OR  congo  OR  "Democratic Republic of the Congo"  OR  "Equatorial Guinea"  OR  gabon  OR  "Sao Tome and Principe"  OR  "Eastern Africa"  OR  burundi  OR  djibouti  OR  eritrea  OR  ethiopia  OR  kenya )  OR  ALL ( rwanda  OR  somalia  OR  "South Sudan"  OR  sudan  OR  tanzania  OR  uganda  OR  "Southern Africa"  OR  angola  OR  botswana  OR  eswatini  OR  lesotho  OR  malawi  OR  mozambique  OR  namibia  OR  "South Africa"  OR  zambia  OR  zimbabwe  OR  "Africa Western"  OR  benin  OR  "Burkina Faso" )  OR  ALL ( "Cabo Verde"  OR  "Cote d'Ivoire"  OR  gambia  OR  ghana  OR  guinea  OR  "Guinea Bissau"  OR  liberia  OR  mali  OR  mauritania  OR  niger  OR  nigeria  OR  senegal  OR  "Sierra Leone"  OR  togo  OR  afghanistan  OR  bangladesh  OR  bhutan  OR  bolivia  OR  cambodia  OR  comoros  OR  egypt )  OR  ALL ( "El Salvador"  OR  haiti  OR  honduras  OR  india  OR  indonesia  OR  kiribati  OR  "North Korea"  OR  "Kyrgyz Republic"  OR  laos  OR  madagascar  OR  micronesia  OR  moldova  OR  mongolia  OR  morocco  OR  myanmar  OR  nepal  OR  nicaragua  OR  pakistan  OR  philippines )  OR  ALL ( "Solomon Islands"  OR  swaziland  OR  "Syrian Arab Republic"  OR  tajikistan  OR  tunisia  OR  ukraine  OR  uzbekistan  OR  vanuatu  OR  vietnam  OR  palestine  OR  yemen  OR  libya  OR  algeria  OR  armenia  OR  azerbaijan  OR  belarus  OR  belize  OR  "Bosnia and Herzegovina" )  OR  ALL ( brazil  OR  colombia  OR  "Costa Rica"  OR  cuba  OR  dominica  OR  "Dominican Republic"  OR  ecuador  OR  fiji  OR  georgia  OR  grenada  OR  guatemala  OR  guyana  OR  honduras  OR  iran  OR  iraq  OR  jamaica  OR  jordan  OR  kiribati  OR  kosovo  OR  "Lao People's Democratic Republic" )  OR  ALL ( lebanon  OR  macedonia  OR  malaysia  OR  maldives  OR  "Marshall Islands"  OR  mexico  OR  paraguay  OR  peru  OR  "Russian Federation"  OR  samoa  OR  serbia  OR  "Sri Lanka"  OR  "Saint Lucia"  OR  "Saint Vincent and the Grenadines"  OR  suriname  OR  thailand  OR  "Timor Leste" )  OR  ALL ( turkey  OR  tonga  OR  turkmenistan  OR  tuvalu  OR  vanuatu  OR  venezuela  OR  "West Bank and Gaza"  OR  "Federated States of Micronesia"  OR  moldova  OR  mongolia  OR  montenegro  OR  myanmar  OR  nauru  OR  nicaragua ) )  AND  DOCTYPE ( ar ) |
| --- |

**CINAHL (27)**

| (Reason* OR Factor* OR Motive* OR Driver* OR determinant*) AND ( "antibacterial agent*" OR antibacteria* OR "antibiotic agent* " OR antibiotic OR "antimicrobial agent" OR antimicrobia* ) AND ( sale* OR Misuse* OR Overuse* OR Inappropriate OR Irrational OR non-judicious OR "Self-medication*" OR "self prescri*" OR practice* OR "Self-treatment*" OR management* OR "over the counter*" OR dispens* OR "without prescription*" OR provision* OR "non prescri" ) AND ( "community pharmac*" OR "Drug store*" OR "Drug vendor*" OR "Drug shop*" OR "Retail Pharmac*" OR "Community Pharmacist*" OR Druggist* OR "Pharmacy technician*" OR "Pharmacy personnel*" OR "Pharmacy assistant*" OR "community pharmacy service*" ) AND ( "Central Africa" OR Cameroon OR "Central African Republic" OR Chad OR Congo OR "Democratic Republic of the Congo" OR "Equatorial Guinea" OR Gabon OR "Sao Tome and Principe" OR "Eastern Africa" OR Burundi OR Djibouti OR Eritrea OR Ethiopia OR Kenya ORRwanda OR Somalia OR "South Sudan" OR Sudan OR Tanzania OR Uganda OR "Southern Africa" OR Angola OR Botswana OR Eswatini OR Lesotho OR Malawi OR Mozambique OR Namibia OR "South Africa" OR Zambia OR Zimbabwe OR "Africa Western" OR Benin OR "Burkina Faso" OR "Cabo Verde" OR "Cote d'Ivoire" OR Gambia OR Ghana OR Guinea OR "Guinea Bissau" OR Liberia OR Mali OR Mauritania OR Niger OR Nigeria OR Senegal OR "Sierra Leone" OR Togo OR Afghanistan OR Bangladesh OR Bhutan OR Bolivia OR Cambodia OR Comoros OR Egypt OR "El Salvador" OR Haiti OR Honduras OR India OR Indonesia OR Kiribati OR "North Korea" OR "Kyrgyz Republic" OR Laos OR Madagascar OR Micronesia OR Moldova OR Mongolia OR Morocco OR Myanmar OR Nepal OR Nicaragua OR Pakistan OR Philippines OR "Solomon Islands" OR Swaziland OR "Syrian Arab Republic" OR Tajikistan OR Tunisia OR Ukraine OR Uzbekistan OR Vanuatu OR Vietnam OR Palestine OR Yemen OR Libya OR Algeria OR Armenia OR Azerbaijan OR Belarus OR Belize OR "Bosnia and Herzegovina" ORBrazil OR Colombia OR "Costa Rica" OR Cuba OR Dominica OR "Dominican Republic" OR Ecuador OR Fiji OR Georgia OR Grenada OR Guatemala OR Guyana OR Honduras OR Iran OR Iraq OR Jamaica OR Jordan OR Kiribati OR Kosovo OR "Lao People's Democratic Republic" OR Lebanon OR Macedonia OR Malaysia OR Maldives OR "Marshall Islands" OR Mexico OR Paraguay OR Peru OR "Russian Federation" OR Samoa OR Serbia OR "Sri Lanka" OR "Saint Lucia" OR "Saint Vincent and the Grenadines" OR Suriname OR Thailand OR "Timor Leste" ORTurkey OR Tonga OR Turkmenistan OR Tuvalu OR Vanuatu OR Venezuela OR "West Bank and Gaza" OR "Federated States of Micronesia" OR Moldova OR Mongolia OR Montenegro OR Myanmar OR Nauru OR Nicaragua ) |
| --- |

**Google Scholar (46):** used the above search strategy

**Hand searching (2):** relevant articles reference check
