# Supplementary material for: No prescription? No problem: drivers of non-prescribed sale of antibiotics among community drug retail outlets in low and middle income countries: a systematic review of qualitative studies
Source: BMC Public Health. 2021 Jun 3;21:1056. doi: 10.1186/s12889-021-11163-3 (PMC8173982; doi:10.1186/s12889-021-11163-3)
Supplement: Supplementary file 2 — Additional file 2:. Quality assessment of the included studies [file 12889_2021_11163_MOESM2_ESM.docx]

**No prescription? No problem: Drivers of non-prescribed sale of antibiotics among community drug retail outlets in low and middle income countries: a systematic review of qualitative studies.**

Sewunet Admasu Belachew^1, 2*^, Lisa Hall^1^, Daniel Asfaw Erku^3^**,** Linda A Selvey^1^

^1^School of Public Health, The University of Queensland, 288 Herston Rd, Herston, Qld 4006, Australia

^2^School of Pharmacy, Faculty of Medicine and Health Sciences, University of Gondar, Ethiopia

^3^Centre for Applied Health Economics, School of Medicine & Menzies Health Institute Queensland, Griffith University, Queensland, Australia.

***Corresponding author**

Sewunet Admasu Belachew

School of Public Health, The University of Queensland, 288 Herston Rd, Herston, Qld 4006, Australia

Email: [s.admasubelachew@uq.edu.au](mailto:s.admasubelachew@uq.edu.au)

**Authors’ email address**

Linda A Selvey: [l.selvey@uq.edu.au](mailto:l.selvey@uq.edu.au)

Lisa Hall: [l.hall3@uq.edu.au](mailto:l.hall3@uq.edu.au)

Daniel Asfaw Erku: [d.erku@griffith.edu.au](mailto:d.erku@griffith.edu.au%20)

**JBI Critical Appraisal Checklist for Qualitative studies**

| **S.N** | | **Criteria** | **S1** | **S2** | **S3** | **S4** | **S5** | **S6** | **S7** | **S8** | **S9** | **S10** | **S11** | **S12** |
| --- | --- | --- | --- | --- | --- | --- | --- | --- | --- | --- | --- | --- | --- | --- |
| 1 | Is there congruity between the stated philosophical perspective and the research methodology? | | N | N | Y | Y | Y | Y | Y | Y | NA | Y | Y | Y |
| 2 | Is there congruity between the research methodology and the research question or objectives? | | Y | Y | Y | Y | Y | Y | Y | Y | Y | Y | Y | Y |
| 3 | Is there congruity between the research methodology and the methods used to collect data? | | Y | Y | Y | Y | Y | Y | Y | Y | Y | Y | Y | Y |
| 4 | Is there congruity between the research methodology and the representation and analysis of data? | | Y | Y | Y | Y | Y | Y | Y | Y | N | Y | Y | Y |
| 5 | Is there congruity between the research methodology and the interpretation of the result | | Y | Y | Y | Y | Y | Y | Y | Y | Y | Y | Y | Y |
| 6 | Is there a statement locating the researcher culturally or theoretically? | | N | N | N | N | N | N | N | N | N | N | N | N |
| 7 | Is the influence of the researcher on the research, and vice- versa, addressed? | | N | N | N | N | N | N | N | N | N | N | N | N |
| 8 | Are participants, and their voices, adequately represented? | | Y | Y | Y | Y | Y | Y | Y | N | Y | N | Y | Y |
| 9 | Is the research ethical according to current criteria or, for recent studies, and is there evidence of ethical approval by an appropriate body? | | Y | Y | Y | Y | Y | Y | Y | Y | Y | N | Y | Y |
| 10 | Do the conclusions drawn in the research report flow from the analysis, or interpretation, of the data? | | Y | Y | Y | Y | Y | Y | Y | Y | Y | Y | Y | Y |
|  | Total score (max 10) | | 7 | 7 | 8 | 8 | 8 | 8 | 8 | 7 | 6 | 6 | 8 | 8 |

**N:** no **Y:** yes **NA:** Not applicable **U:** unclear

**Studies (S):**

**S 1**: Non-prescribed sale of antibiotics for acute childhood diarrhoea and upper respiratory tract infection in community pharmacies: a 2 phase mixed-methods study

**S 2:** Exploration of over the counter sales of antibiotics in community pharmacies of Addis Ababa, Ethiopia: pharmacy professionals’ perspective

**S 3:** Exploring the reasons why pharmacists dispense antibiotics without prescriptions in Khartoum state, Sudan

**S 4**: What motivates antibiotic dispensing in accredited drug dispensing outlets in Tanzania? A qualitative study

**S 5**: A qualitative analysis of pharmacists’ attitudes and practices regarding the sale of antibiotics without prescription in Syria

**S 6**: Factors associated with inappropriate dispensing of antibiotics among non-pharmacist pharmacy workers

**S 7.** What drives inappropriate antibiotic dispensing? A mixed-methods study of pharmacy employee perspectives in Haryana, India

**S 8**. Antibiotic sales in rural and urban pharmacies in northern Vietnam: an observational study

**S 9**: Irrational use of antibiotics and role of the pharmacist: an insight from a qualitative study in New Delhi, India

**S 10**: Sale of Antibiotics without Prescriptions in Alexandria, Egypt

**S 11**: Antimicrobial dispensing practices and determinants of antimicrobial resistance: a qualitative study among community pharmacists in Pakistan

**S 12**: Determinants of non-prescription antibiotic dispensing in Chinese community pharmacies from socio-ecological and health system perspectives
